# Perspective on applicability of data-driven machine learning computational new approach methodologies for hazard identification in chemicals risk assessment

**DOI:** 10.1186/s13321-026-01184-8

**Published:** 2026-03-26

**Authors:** Geven Piir, Sulev Sild, Olga Tcheremenskaia, Emma Di Consiglio, Jörgen Henriksson, Agnieszka Gajewicz-Skretna, Enrico Mombelli, Alessandra Roncaglioni, Uko Maran

**Affiliations:** 1 Institute of Chemistry, University of Tartu, Ravila 14a, 50411 Tartu, Estonia; 2 Department of Environment and Health, National Institute of Health (ISS), Rome, Italy; 3 Swedish Chemicals Agency, Sundbyberg, Sweden; 4 Laboratory of Environmental Chemoinformatics, Faculty of Chemistry, University of Gdansk, Gdansk, Poland; 5 Department of Physical Chemistry, Faculty of Chemistry, Gdansk University of Technology, Gdansk, Poland; 6 French National Institute for Industrial Environment and Risks (INERIS), Verneuil-en-Halatte Parc Technologique ALATA, BP 2, Verneuil-en-Halatte, France; 7 Department of Environemntal Health Sciences, Istituto Di Ricerche Farmacologiche Mario Negri IRCCS, Milan, Italy

## Abstract

Machine Learning (ML) and Artificial Intelligence (AI) approaches have potential to make better-informed decisions in chemical hazard identification while reducing animal testing. Their application in the context of New Approach Methodologies (NAMs) for Hazard Identification in Chemicals Risk Assessment (CRA) is challenging due to the limited knowledge, lack of experience, and uncertainty related to the use of these approaches. Therefore, to facilitate ML and AI approaches' potential acceptance for regulatory use, better standardization, guidelines for transparent reporting, validation, and frameworks are needed to understand their accessibility, verifiability, and usefulness criteria for predictions. An extensive literature review on the availability of ML and AI based NAMs for chemical hazard identification was conducted, focusing primarily on human health endpoints: specific target organ toxicity (STOT), genotoxicity and carcinogenicity, endocrine disruption, skin sensitization, developmental and reproductive toxicity (DART), and repeated dose or chronic toxicity. Nearly 2300 scientific articles were reviewed, and 274 publications with ML-QSAR models revealed that 60.9% of the models described in the scientific literature turned out to be non-usable, 21.9% were potentially usable, and 17.2% were directly usable, i.e., had available software solutions. By endpoint, the skin sensitization is best covered with the ML-QSAR models, followed by endocrine disruption, genotoxicity, and carcinogenicity models. The most derived ML-QSAR models are tree-based models such as random forests, and analogues, followed by artificial neural networks and support vector machine models, with other models being used to a lesser extent. The literature analysis led to a framework that helps model users to identify potentially suitable models for use in a regulatory context. In addition, the framework could help model developers better understand the expectations of model users in a regulatory context and use the framework as a reference when publishing their models, ensuring greater transparency, alignment with regulatory needs, and facilitating future acceptance.

**Supplementary Information:**

The online version contains supplementary material available at 10.1186/s13321-026-01184-8.

## Introduction

Hazard assessment is the cornerstone of Chemicals Risk Assessment (CRA) and consists of hazard identification and hazard characterization [1–6]. Hazard identification determines whether a substance can cause adverse effects and identifies the specific types of harm it may pose to human health or the environment, while hazard characterization provides a quantitative description of the nature and severity of the adverse health effects a chemical may cause (i.e., doses involved and related responses). Mainly, hazard identification will be considered within the framework of this article, although risk characterization is also covered to a lesser extent.

Traditionally, hazard identification relied heavily on empirical and animal testing data [7, 8]. However, ethical concerns, logistical challenges, cost, and the growing demand for efficient and more reliable risk assessment methods have spurred the advancement of alternative testing approaches [9–11]. The emergence of New Approach Methodologies (see next section) represents a paradigm shift in hazard identification, offering more scientifically robust, efficient, cost-effective, and ethical alternatives to traditional methods. NAMs encompass a diverse array of non-animal methodologies, including computational modelling, high-throughput screening assays, and in vitro testing, which enables the prediction of chemical hazards based on structural, bioavailability, and bioactivity data [12, 13]. By using NAMs, regulatory agencies and researchers may overcome the limitations of traditional hazard identification methods, such as reliance on animal testing, enabling efficient assessment of large numbers of chemicals and gaining valuable insights into the underlying mechanisms of chemical toxicity.

Machine learning is becoming an increasingly essential component of computational NAMs (see section on machine learning), which are emerging as tools for identifying chemical hazards. Publishing ML models in the scientific literature makes them discoverable and accessible for a wider audience. This prompted the aim of this study to explore the scientific literature and determine the accessibility and applicability of ML models for hazard identification in the frame of CRA. Current research covered recently published computational NAM models targeting various human health and, in a few cases, environmental endpoints relevant to the “Partnership for the Assessment of Risks from Chemicals (PARC)” project. Based on this exploratory analysis, the main barriers to the application of ML models were discussed, and strategies are proposed to increase the use of computational NAMs in CRA by providing a framework for the effective use of the models.

###  Computational new approach methodologies

Classical chemical risk assessment relies on animal test data. At the same time, there is intense pressure to abandon animal testing in every chemical-related field [14]. As a result, there is a growing movement across research and regulatory fields to reduce, replace, and refine animal testing in line with the 3Rs principle [11, 15]. NAM is a generalisation of any chemical testing methods that give information about chemical hazards without using animals for testing. The nature of NAMs has been articulated in many ways by different organizations during the evolution of this term: OECD [16], ECHA [17], EFSA [18–20], US EPA [21–23], ICCVAM [24], Health Canada [25], and UK CoT&FSA [26]. Based on the content of this article and proposal for definition within the EU funded project PARC (task T6.4.2) and possible developments in the future, the authors use the following definition for NAM: *NAMs are defined as any valid and relevant technology, methodology, approach, strategies, or combination thereof that can provide information on chemical hazard, exposure, and risk assessment to reduce, refine or replace animal testing*. Consequently, NAMs can include or are a combination of a wide range of in chemico [27], in vitro [28]*,* and in silico [29] methods. The latter includes computational approaches that can be integrated into tiered testing and assessment workflows (e.g., Integrated Approaches to Testing and Assessment (IATA) and Defined Approach (DA)) for specific endpoints and can be used as a standard test battery to provide toxicological information in the context of hazard, exposure, and risk assessment [30, 31].

The first group of computational NAMs is represented by expert systems that apply existing knowledge and experience in the form of models to identify and explain mechanisms of action, such as the so-called Structure–Activity Relationship (SAR) approach, using structural alerts or multidimensional analogy methods (like Read-across) [32]. The second larger group is made up of so-called mechanism-based systems, i.e., models derived from scientific principles and assumptions about mechanism-based processes and have found application in the form of quantitative Adverse Outcome Pathway (AOP) models, and exposure and, multiple media fate models such as Physiologically Based Kinetic (PBK). While the previous two groups largely use existing experimental information in constructing the computational model, the third group comprises those computational models that focus on a data-driven learning-based approach. The central axis here is an approach that links the structure of a chemical and its properties, the so-called Quantitative Structure–Activity Relationship ((Q)SAR) approach, which currently applies the entire arsenal of ML and Artificial Intelligence (AI) methods. ML and AI have, in turn, found application within the framework of (Q)SAR because they facilitate the analysis of larger and more diverse data sets based on structure, allowing for effective input into the identification of chemical risks within the framework of NAMs.

###  Machine learning

In terms of terminology, ML and AI are often used interchangeably and are not well defined across disciplines [33]. Although associated and overlapping in certain elements, they should be looked at separately because their purposes are different [34]. AI refers to the ability of computer-based systems to simulate human cognitive functions, i.e., the ability to reason, solve problems, discover meanings, generalize, or learn from previous experiences. Today's AI systems run on computers, phones, servers, and other devices, as well as on special-purpose hardware devices (e.g., robots, autonomous cars, and Internet of Things devices). Typical applications of AI include image and speech recognition, text analysis, virtual assistants, fraud detection, medical diagnostics, drug development, environmental monitoring, and risk analysis [35–38]. Based on this, and within the scope of the present publication and work carried out under PARC (task T6.4.2), the following working definition of AI was used: *Artificial Intelligence is the ability of computer-based systems to simulate human cognitive functions (e.g., reason, solve problems, discover meaning, generalize, i.e., learn from past experience). Current leading methods are different versions of Artificial Neural Networks.*

In contrast, the working definition for ML was the following: Machine Learning *is a set of methods that builds models from training samples (e.g., chemical and biological data), to learn from data to create new information. It is using a variety of statistical and predictive analysis methods and their combinations (e.g., Decision Trees, Support Vector Machine, Random Forest, Bayesian networks, Regression Analysis, Artificial Neural Networks, *etc*.).* Although ML is often considered a subset of AI, there are diverging views on this topic, as it is now recognized as a separate field of research and has different goals [39]. Still, both fields are closely related and have overlapping applications. ML, as a field, focuses on methods to create models based on training data, while AI is more focused on simulating human cognitive functions. Some ML methods are inspired by AI but also include many other methods outside the field of AI, e.g., support vector machines, random forests, Bayesian classification, etc. Therefore, when evaluating different artificial intelligence applications, it is important to recognize whether it is genuinely an AI or an ML application. Thus, ML refers to a set of methods used to generate new information, such as predicting a property, while AI is a set of methods designed to make decisions, ideally mimicking human reasoning.

###  Classification of ML approaches

**Fig. 1:**
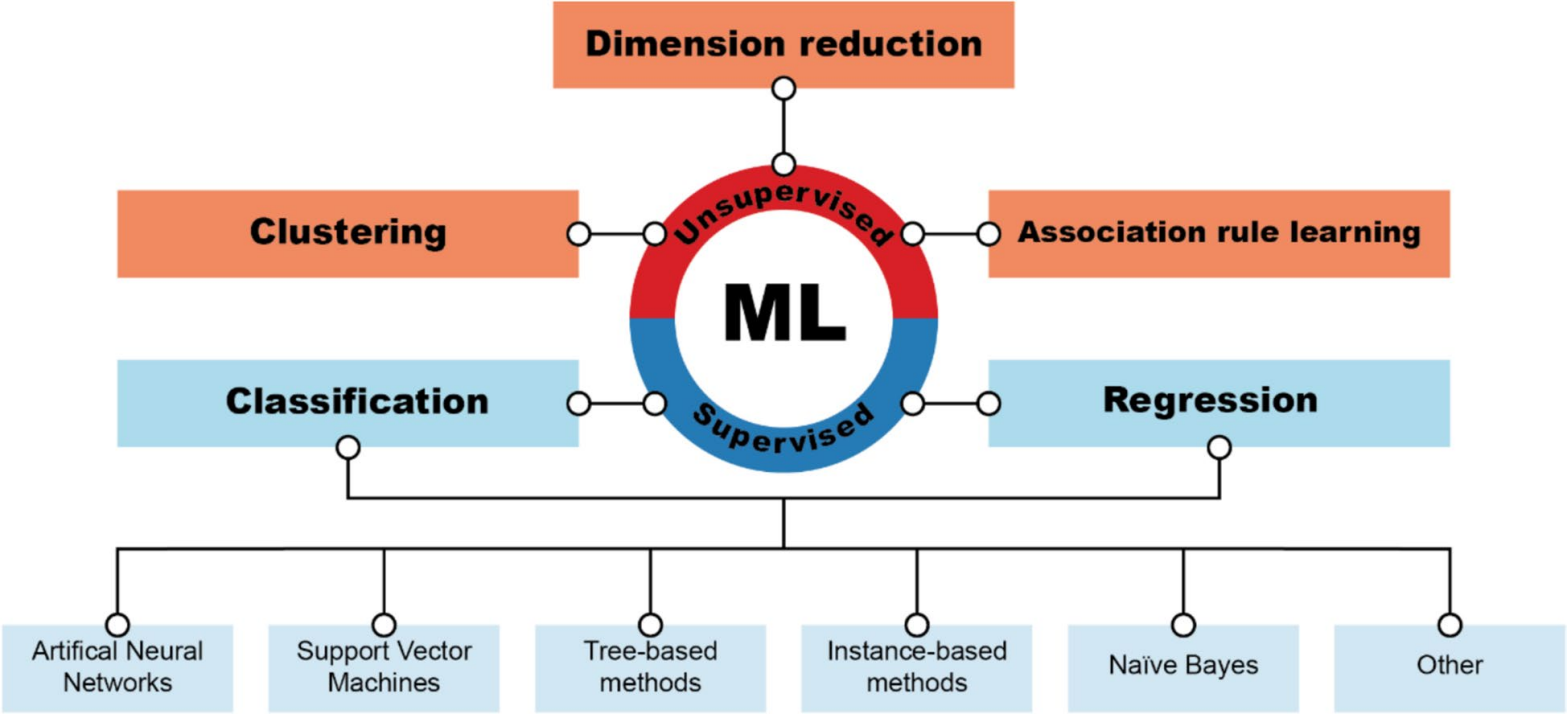
Classification of ML approaches

ML algorithms can be grouped in many ways [40, 41]. Here, ML approaches are divided into unsupervised and supervised learning methods (Fig. 1). The unsupervised learning uses unlabelled data, and three types of methods fit under its roof: clustering, dimension reduction, and association rule learning [42, 43]. It is usually used to find hidden patterns in data, and in the context of (Q)SAR, it is used as a pre-processing step before applying supervised learning methods. For supervised learning methods, the objects in the data are labelled or described with numbers. Therefore, the analysis that can be performed is either classification (e.g., determining whether a chemical is toxic or not) or regression (e.g., determining numerically how toxic a chemical is). CRA relies on such information available on chemicals; therefore, this study focuses on supervised ML methods. Generally, the same algorithms (with slight modifications) can be applied for both analysis types. This review groups those algorithms into six broad groups: artificial neural networks, support vector machines, tree-based methods, instance-based methods, Naïve Bayes methods, and other methods. The final group contains all the techniques that do not fit into the previous groups.

Each ML approach has distinct strengths and limitations that arise from its underlying assumptions and the complexity of the approach. The selection of an appropriate ML method should be guided by the nature of the data, the complexity of the problem, the availability of computational resources, and the interpretability requirements specific to the research question under investigation. Below is a brief introduction to primary ML methods, highlighting their advantages and disadvantages.

Artificial neural networks (ANN) are computational models inspired by biological neural systems. ANNs consist of interconnected layers of neurons that model complex nonlinear relationships through iterative training procedures [44, 45]. Methodologically, ANNs adjust their internal weights using optimization algorithms (e.g., gradient descent) to minimize prediction errors. Their strengths include exceptional predictive accuracy, handling large data sets, and adaptability to diverse problems, including regression, classification, and pattern recognition [46–48]. However, ANNs require extensive training data. Major drawbacks include high computational cost, extensive data requirements to prevent overfitting, sensitivity to network architecture and hyperparameters, and difficulty in interpreting internal model structures, often referred to as “black box” models [48, 49].

Support vector machines (SVMs) are supervised methods for classification and regression tasks that determine the optimal separating hyperplane to maximize the margin between classes or minimize the prediction error, respectively [50, 51]. Their fundamental methodological assumptions are based on linear separability, although nonlinear problems can be solved using kernel functions (e.g., radial basis function, polynomial kernel, or sigmoid kernel) [52]. The advantages of SVMs include robustness to high-dimensional data, effectiveness on small data sets, and flexibility through kernel functions. In addition, SVMs are less prone to overfitting than other algorithms (e.g., decision trees) and less sensitive to noise (i.e., outliers). However, SVMs are inherently difficult to interpret directly due to the high-dimensional transformations, are sensitive to parameter tuning (i.e., kernel type, cost parameter, gamma), and computationally demanding for large datasets [52–54].

Tree-based methods are supervised learning, nonparametric methods that address classification or regression problems by constructing a tree-like structure to make predictions. They employ recursive partitioning of data, using binary splits based on input features to model relationships. These methods efficiently capture complex, linear, and nonlinear relationships [55, 56]. Advantages also include inherent feature importance scoring and resistance to outliers. Decision trees (DTs) are simple and easy to interpret, but prone to overfitting and instability. Ensemble methods, such as random forest (RF) and XGBoost, address these limitations by aggregating the results of multiple trees to improve predictive accuracy, stability, and robustness. RF uses bagging to build uncorrelated trees, significantly reducing variance. XGBoost uses boosting, which iteratively builds new trees to correct the errors of previous trees, optimizing predictive performance. However, ensemble approaches often sacrifice interpretability compared to single trees, become “black box” models, and can require significant computational resources and careful hyperparameter tuning [56–58]].

The k-nearest neighbours (k-NN) algorithm is a nonparametric, instance-based algorithm for classification and regression tasks. It classifies or predicts based on the majority class or the average of its 'k' nearest neighbours [59, 60]. Methodologically, it relies solely on feature similarity measured by distance metrics (e.g., Euclidean distance, Manhattan distance). Its advantages include simplicity, intuitive implementation, and no explicit assumption about the underlying data distribution. This means that it is suitable for linear, nonlinear, and multimodal data distributions. On the downside, k-NN can be computationally intensive, especially for large datasets (due to distance calculations), sensitive to irrelevant features and noise, and the challenge of selecting an appropriate number of neighbours (k) and distance metric. It can also suffer significantly from the curse of dimensionality [61, 62].

Naïve Bayes methods are probabilistic classifiers based on Bayes' theorem with the fundamental "naive" assumption of conditional independence between each pair of features given the value of the class variable [63]. These methods are straightforward, computationally efficient, easily scalable, and perform particularly well on high-dimensional datasets. Naïve Bayes methods also handle missing data and noisy features relatively well. However, the fundamental feature independence assumption rarely holds in practice, limiting their performance and accuracy on datasets with strong feature interdependencies or correlations. Despite this drawback, Naïve Bayes remains robust and effective for many classification tasks [64].

##  Methods

###  Selection of endpoints

EU regulatory frameworks require specific toxicological data/information to address defined safety endpoints, as mandated by a broad set of regulations, such as REACH (EC) No 1907/2006, the Food Contact Materials regulation (EC) No 1935/2004, the Plant Protection Products regulation (EC) No 1107/2009b, the Cosmetics regulation (EC) No 1223/2009, the Classification, labelling and packaging (CLP) regulation (EC) No 1272/2008, the Biocides regulation (EC) No 528/2012, and strategic initiatives, such as EU chemicals strategy for sustainability (COM/2020/667 final), and the European Green Deal (COM/2019/640 final).

The toxicological endpoints selected in the PARC project were chosen based on regulatory priorities, scientific gaps, and the potential for methodological innovation. The rationale behind their selection includes several aspects such as (i) alignment with current regulatory needs, (ii) potential use of NAMs, (iii) public health relevance, (iv) scientific gaps and lack of validated approaches, and (v) shift toward mechanism-based risk assessment.

PARC aims to address critical regulatory data gaps by promoting a transition from an endpoint-based animal testing strategy to a more mechanistic-based NAMs testing strategies. These strategies target complex toxicological endpoints such as non-genotoxic carcinogenicity, immunotoxicity, specific organ toxicity, DNA damage, endocrine disruption, and (developmental) neurotoxicity.

Based on the endpoints prioritized by work packages five and six of PARC, a list of endpoints has been developed to guide the literature search conducted in this study**.** These endpoints offer strong potential for the development and application of in silico approaches (e.g., QSAR models, machine learning) and mechanistic frameworks like AOPs. The selected endpoints are linked to chronic diseases and emerging health concerns such as neurodevelopmental disorders in children, metabolic dysfunctions, and immune-related conditions. PARC aims to fill these gaps by developing and validating innovative tools.

###  Literature search strategy

A literature search was carried out using the subscription-based Web of Science (“Web of Science Core Collection” search over All Fields). Publications for the following endpoints were searched: specific organ toxicity, such as liver (hepatic), kidney (renal), heart (cardio), pulmonary (lung) toxicity, and neurotoxicity, genotoxicity, carcinogenicity, endocrine disruption, skin sensitization, developmental and reproductive toxicity, and chronic (repeated dose) toxicity. For the literature search, endpoint-specific terms were used systematically and were combined with the term “machine learning” or “QS*R” (see Supplementary Information for search links). The search covered the years from 2018 to 2023. The choice of 2018 as the starting year was selected because it marks the beginning of the promotion of the NAMs in this field. The search results were manually checked to find articles corresponding to the endpoint of concern and held original QSAR models developed with machine learning (see Supplementary Information). In all cases, the frequency of occurrence of different machine learning methods was also recorded.

###  Model accessibility criteria

**Fig. 2:**
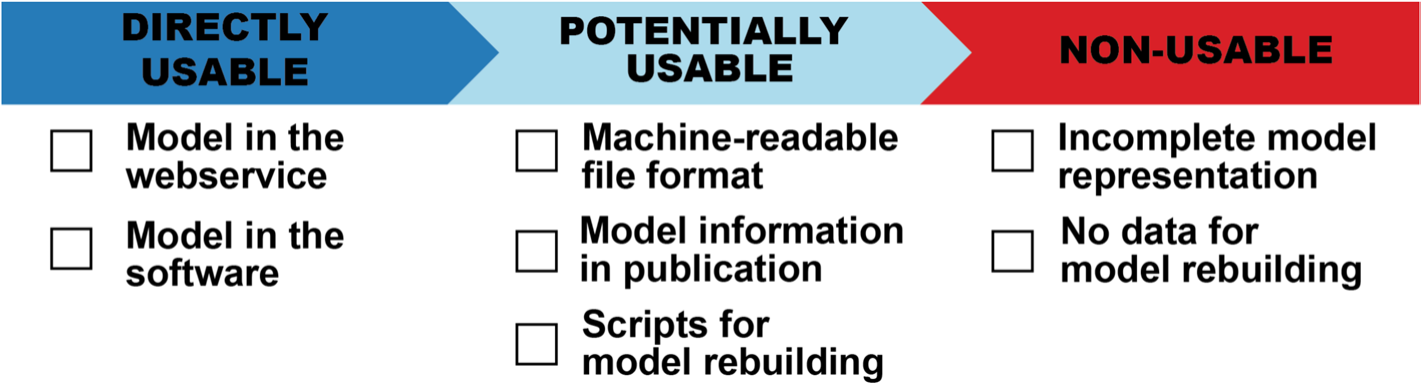
Criteria for model usability

The review took a detailed look at the models’ accessibility. It divided them into three categories, based on how easily those models can be used: (a) directly usable, (b) potentially usable, and (c) non-usable. The checklist (Fig. 2) has the criteria used to assess whether the model can be used. Models were considered directly usable when integrated into a web service or published as standalone software. It must be emphasised that only the model's ability to make predictions was assessed, and its suitability for different tasks (e.g., screening) was not assessed. According to the best practices of model representation [65], see check-list there], that correspond to the OECD QSAR validation principles (particularly Principle 2) [66, 67], a potentially usable model must be published in a machine-readable file format (e.g., PMML, ONNX), or all the relevant information that allows usage of the model is included. For example, the publication includes complete DT; ANN was published with architecture, weights, and activation functions; SVM had all the parameters together with support vectors. Such models, together with publications that had information about data and scripts for model rebuilding, were put in the second category. However, whether the potentially usable models were reproducible was not checked as part of the analysis. The third category held publications that did not have enough data for model rebuilding nor presented the model in a directly usable manner.

##  Results

**Fig. 3:**
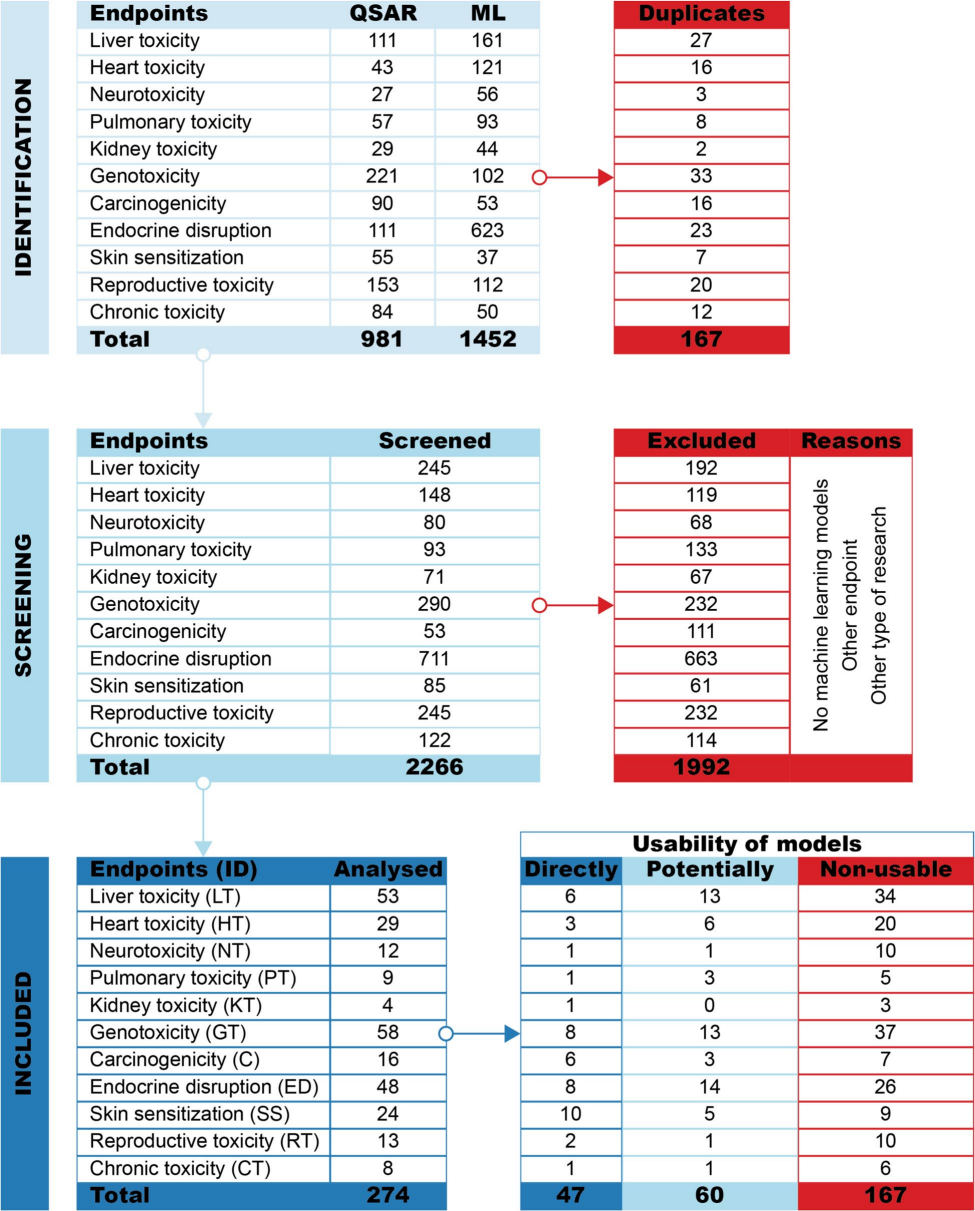
Results of the literature search

**Fig. 4:**
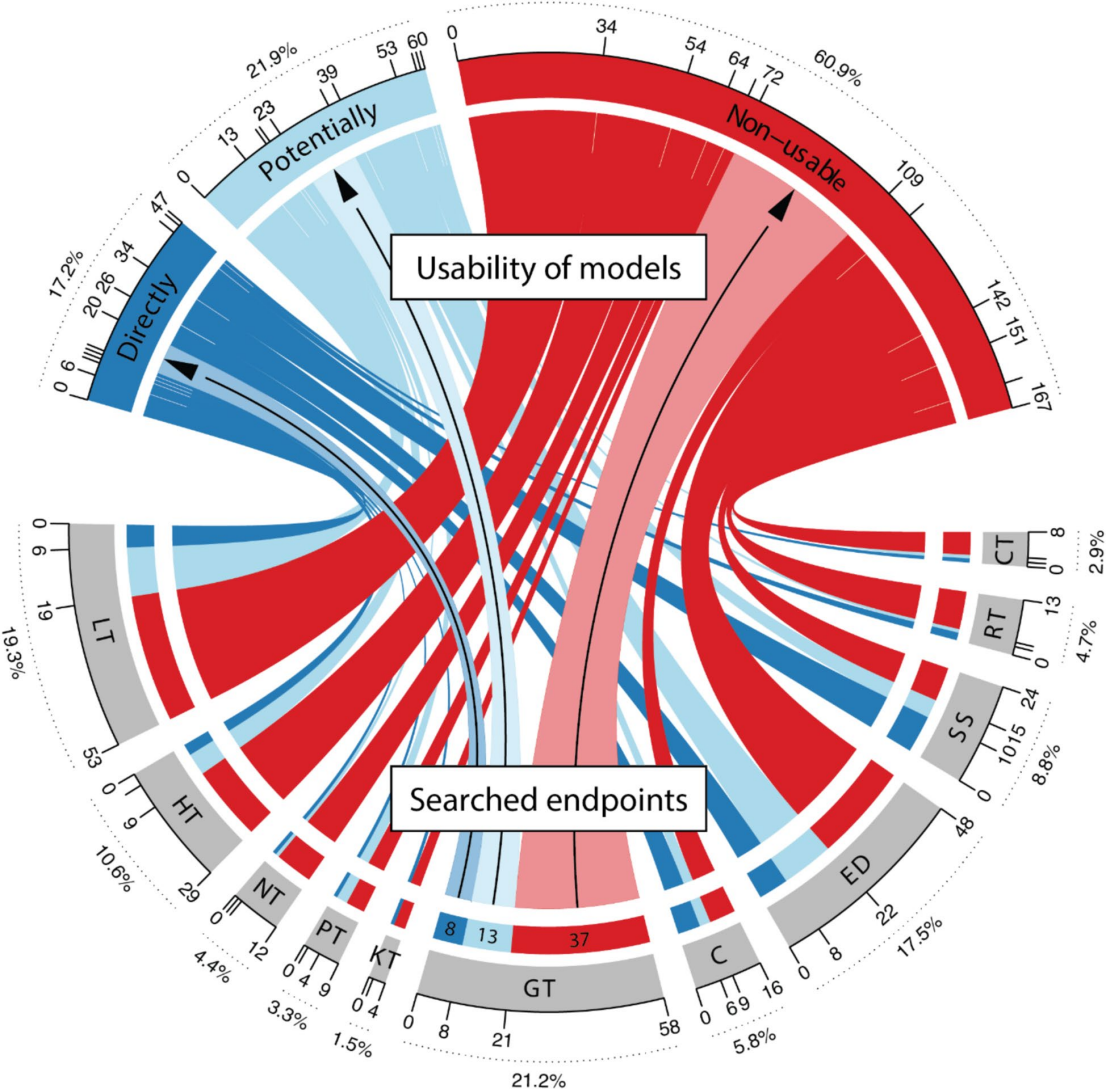
Summary of search results as a Circos plot

The literature search for eleven toxicological endpoints to identify (Q)SAR and ML models resulted in 2433 hits (Fig. 3, identification). Since (Q)SAR and ML models were searched separately, there was an overlap (167) between hits. A total of 2266 articles were screened (Fig. 3, screening), of which 1992 were excluded because they either lacked models (e.g., existing models were used to predict properties for compounds that were synthesized in publication), focused on different endpoints (e.g., membrane permeability), or described unrelated types of research (e.g., patient analysis). In total, 274 articles referring to ML models remained for the final review (Fig. 3, included). Of these, 260 were unique, while some articles addressed multiple endpoints. Interestingly, most articles (236) focused on building and explaining classification models. Only regression models were developed in 22 papers, and 16 dealt with classification and regression. The results are summarised in Fig. 3 (usability of models) and visualised in Fig. 4

The visualisation of the results (Fig. 4) provides an endpoint-based overview of the usability of models from articles. In total, there were 47 articles where models were directly usable (dark blue), 60 cases where models were potentially usable (light blue), and 167 cases where models were non-usable (red). Connections from each endpoint (lower half) show how much each of them contributes to the overall results of the literature search (upper half). For example, genotoxicity (see Fig. 4, GT) was modelled 58 times, and directly usable models were published 8 times, potentially usable 13 times, and non-usable 37 times. In summary, over 17% of the articles contained directly usable models that were implemented in the software or web services (Table 1), and nearly 22% of the models published in the articles were potentially usable because they had access to the code (Table 2). The remaining 61% of articles did not present ML models in a usable way. On a positive note, the situation has improved considerably within the last five years. Our earlier review [65] showed that only 10% of the other than multiple linear regression models were potentially usable. It is worth mentioning that in many publications, existing (both freely available and commercial) models were used to predict some endpoints. A typical use case was the prediction of genotoxicity and carcinogenicity of chemicals that were synthesized and reported in a publication.

**Table 1:** Directly usable models available through a freely accessible web service or software

Endpoint ID	Name (Web Service or SoftWare): URL	References
CT, C	VEGA (SW): https://www.vegahub.eu	[68, 69]
KT, GT, C, RT	SApredictor (WS): http://www.sapredictor.cn	[70, 71]
LT, GT, C	ProTox (WS): https://tox-new.charite.de/protox_II/	[72]
LT	SiliS-PTOXRA (WS): http://tomocomd.com/apps/ptoxra	[73]
LT	Vienna-LiverTox (WS): https://pharminfo.univie.ac.at/services/vienna-livertox	[74]
LT	DILI-Stk (WS): http://ssbio.cau.ac.kr/software/dili	[75]
LT, SS, GT	MUDRA (WS): https://chembench.mml.unc.edu/mudra/	[76]
LT, GT, C	VenomPred (WS): http://www.mmvsl.it/wp/venompred2/	[77]
SS	SkinDoctorCP (WS): https://nerdd.univie.ac.at/skin-doctor-cp	[78]
SS	admetSAR (WS): http://lmmd.ecust.edu.cn/admetsar2/	[79]
SS	PreS/MD (WS): https://presmd.mml.unc.edu	[80]
SS	SkinSensDB (WS): https://cwtung.nhri.edu.tw/skinsensdb/	[81–83]
SS	PRED-SKIN (WS): http://predskin.labmol.com.br/	[84]
SS	STopTox (WS): https://stoptox.mml.unc.edu	[85]
SS	SkinSensitizerCalculator (SW): https://sites.google.com/jadavpuruniversity.in/dtc-lab-software/home?pli=1#h.4ulkpu4lhexc	[86]
GT	LAZAR (WS): https://lazar.in-silico.ch	[87]
GT, C	VenomPred (WS): http://www.mmvsl.it/wp/venompred/	[88]
GT, C	MicotoXilico (WS): https://chemopredictionsuite.com/MicotoXilico	[89]
GT	ADMETopt2 (WS): http://lmmd.ecust.edu.cn/admetsar2/admetopt2/	[90]
RT	TIRESIA (WS): http://tiresia.uniba.it/	[91]
NT	DINeuroTpredictor (WS): http://dineurot.sapredictor.cn	[92]
ED	NN^a^ (SW): https://figshare.com/articles/code/estrogen_receptor_Bayesian_Machine_learning_models/6267263	[93]
ED	Predictor EU-REACH endpoints (WS): http://infochim.u-strasbg.fr/cgi-bin/predictor_reach.cgi	[94]
ED	ToxicityPredictor (WS): http://mmi-03.my-pharm.ac.jp/tox1/	[95]
ED	NR-TOXPRED (WS): http://nr-toxpred.cchem.berkeley.edu/predict	[96]
ED	QsarDB.org (WS): https://qsardb.org/repository/handle/10967/236	[97]
ED	OPERA (WS & SW): https://github.com/NIEHS/OPERA, https://comptox.epa.gov/dashboard/	[98]
ED	EDC-Predictor (WS): http://lmmd.ecust.edu.cn/edcpred/	[99]
ED	EDTox (WS): http://www.edtox.fi	[100]
HT	CardPred (WS): http://ssbio.cau.ac.kr/CardPred	[101]
HT	cardioToxCSM (WS): https://biosig.lab.uq.edu.au/cardiotoxcsm	[102]
HT	ICDrug (WS): http://www.icdrug.com/ICDrug/T	[103]
PT	ProfhEX (WS): https://profhex.exscalate.eu	[104]

^a^ No Name (NN)

**Table 2:** Scripts for potentially usable models

Endpoint ID	URL	References
CT	https://github.com/ifyoungnet/NOAEL	[105]
LT	https://github.com/YanLabAI/SpectraTox	[106]
LT	https://github.com/anikaliu/CAMDA-DILI	[107]
LT	https://github.com/hurlab/CAMDA-Challenge-2020-Drug-Induced-Liver-Injury	[108]
LT	https://github.com/volkamerlab/CPrecalibration_manuscript_SI	[109]
LT	https://github.com/TingLi2016/DeepDILI	[110]
LT	https://github.com/TX-2017/machine-learning	[111]
LT	https://github.com/dreadlesss/Hepatotoxicity_predictor	[112]
LT	https://github.com/CSU-QJY/GeoDILI	[113]
LT	https://github.com/sslim0814/SSM	[114]
SS	https://figshare.com/articles/code/Naive_Bayes_Skin_Sensitization_Model/5758644	[115]
SS	https://github.com/Wenying-Yu-Lab/HOLTEP-CPU	[116]
SS, GT	https://github.com/marinaglr/metabio	[117]
GT	http://www.sbx.mutapred.com.s3-website-ap-northeast-1.amazonaws.com	[118]
GT	https://github.com/12rajnish/mutagenicity-prediction	[119]
GT	https://github.com/ntua-unit-of-control-and-informatics/MWCNTs https://github.com/ntua-unit-of-control-and-informatics/SPIONs)	[120]
GT	https://github.com/VirginiaSabando/MTL_DNN_Ames	[121]
GT	https://github.com/chiakangZacHung/Gini_Hung-QSAR_GCN/tree/main	[122]
GT	https://github.com/mowal/Imputation_Paper	[123]
GT	https://github.com/SamuelFeeney/Multiple-instance-learning-improves-Ames-mutagencity-prediction-for-problematic-molecular-species	[124]
GT	https://github.com/luiraym/gmtames	[125]
GT	https://github.com/TingLi2016/DeepAmes	[126]
C	https://github.com/TingLi2016/DeepCarc	[127]
C	https://github.com/HuazhouZhang/CarcGC	[128]
NT	https://github.com/LCSB-DVB/Monzel_2020	[129]
ED	https://gitlab.fbk.eu/toxpred/DL4Tox	[130]
ED	https://github.com/DGadaleta88/TPO_QSAR	[131]
ED	https://github.com/Feesterra/Conformal_THS	[132]
ED	https://github.com/vittoriofortino84/EDTOX	[133]
ED	https://michem.unimib.it/download/data/ar-binding-compara-project/	[134]
ED	https://zenodo.org/record/7310722#.Y5CqWnbMJXs	[135]
ED	https://github.com/Agnes-L/ML-classifiers-with-ADSAL	[136]
ED	https://github.com/volkamerlab/knowtox_manuscript_SI	[137]
HT	https://github.com/PDelre93/hERG-QSAR	[138]
HT	https://github.com/Liu-Lab-Lnu/MDFP-hERG	[139]
HT	https://github.com/ChengF-Lab/deephERG	[140]
HT	https://zenodo.org/records/7551783	[141]
HT	https://github.com/AI-amateur/DMPNN-hERG	[142]
HT	https://github.com/NIDA-IRP-CCMB/QSAR_DAT-hERG	[143]
PT	https://zenodo.org/records/6619307	[144]
PT	https://zenodo.org/records/4660115	[145]

The analysis of ML approaches revealed that various complex algorithms were utilised for quantitative and qualitative structure–activity relationships. As discussed above, ML algorithms can be grouped differently. Here, ML algorithms were divided into six wide-scoped groups based on general algorithm structure: artificial neural networks (111 publications), support vector machines (127), tree-based methods (201), instance-based methods (84), Naïve Bayes methods (56), and other methods (60). Table 3 shows how models from those groups fit into model accessibility criteria. Independent of their type and architecture, artificial neural networks were assembled, and they formed the most diverse group, including over 15 different neural nets. Tree-based methods, such as decision trees, random forests, gradient, and adaptive boosting, formed the largest group. The instance-based group included methods like read across and k-nearest neighbours. All the algorithms that did not fit under the first five groups were placed in the other group.

**Table 3:** Usability of models grouped by algorithm types

Algorithm	Total	Usable (%)	Potentially usable (%)	Non-usable (%)
ANN	111	16 (14)	20 (18)	75 (68)
SVM	127	23 (18)	24 (19)	80 (63)
Trees	201	39 (19)	44 (22)	118 (59)
Instance	84	17 (20)	14 (17)	53 (63)
NB	56	10 (18)	9 (16)	37 (66)
Other	60	12 (20)	18 (30)	30 (50)
Total	639	117 (18)	129 (20)	393 (62)

###  Specific target organ toxicity (STOT)

This subsection covers five searched endpoints (liver toxicity, heart toxicity, neurotoxicity, pulmonary toxicity, and kidney toxicity). Together, they were covered by 108 articles, of which 36 may be considered usable. However, the number of published articles per endpoint varied significantly (Table 1).

Liver toxicity (LT) had the most hits in search results. All 53 articles described classification models and addressed different endpoints of liver toxicity. The links in the Fig. 4 show that only six of the 53 articles were directly usable (Table 1, Endpoint LT), and thirteen articles had potentially usable models because they provided enough information to reuse them (Table 2, Endpoint LT). Of the articles reviewed, scripts were uploaded to an external repository nine times (Table 2) and in four cases, this information was included in the supplementary material or in the article [146–149]. Unfortunately, more than 62% (34 articles) had incomplete descriptions of models to be considered usable.

In this category, the second-highest number of articles (29) were related to heart toxicity (HT). Of those, 21 publications developed only classification models, five used pure regression models, and three articles used both kinds of models. Only three of the 29 articles reviewed provided directly usable models (Table 1, Endpoint HT), while code was provided in six publications (Table 2, HT). As such, models from 18 articles were not usable.

The search for other endpoints in this group resulted in 25 hits. Neurotoxicity (NT) had the most articles (12) with ML models and was followed by pulmonary toxicity (PT) with nine, and kidney toxicity (KT) with four articles. The models appearing in the articles were described with little detail. Each endpoint had one directly usable model (Table 1, Endpoints NT, PT, KT), three had scripts in the external repository (Table 2, Endpoints NT, PT), and one had a model in the publication [150].

###  Genotoxicity and carcinogenicity

Out of all the endpoints searched, genotoxicity (GT) had the highest number of articles (58) featuring models. Almost all of these were classification models, with only two proposing regression models. Although many articles with ML models were found, most (37) were unusable. Direct access for the prediction service was provided eight times (Table 1, Endpoint GT), and ten times, the code for models was accessible via an external repository (Table 2, Endpoint GT). Finally, three publications published details for the model in supplementary information or the article [151–153].

The literature search yielded 16 articles on carcinogenicity (C), seven of which had unusable models. All articles presented classification models, while additional regression models were present in three articles. Models were implemented into the software on one occasion, and five other models have been implemented into web services (Table 1, Endpoint C). The scripts were uploaded into the repository twice (Table 2, C) and once the decision tree was present in the article [154].

###  Endocrine disruption

In the search for models of endocrine disruption (ED) effects, estrogen, androgen, and thyroid modalities were considered. The literature search and analysis yielded 48 articles with ML models. Of the articles with models, 26 had an incomplete description of models and needed more detailed information to be usable. However, this category had the most models that could be usable. The models from eight articles were directly usable (Table 1, Endpoint ED), and an additional 14 were potentially usable. From those, eight publications used external repositories to preserve model-building scripts (Table 2, ED). Model-building scripts or the model itself were included in the Supplementary Information in three publications [155–157], and three times, the model was published within the article, making those models potentially usable [158–160].

###  Skin sensitization

The literature search revealed 24 articles with models for skin sensitization (SS); for this endpoint, the largest number of models from articles (10) were directly usable (Table 1, Endpoint SS). From those, one model was inside a stand-alone software, and others were integrated into web services. However, three publications pointed to the same web service. In addition to explicit access, three articles provided workflows to reuse models in external repositories (Table 2, SS) or provided scripts as Supplementary Information [161, 162]. All the other articles did not represent models in a usable manner.

###  Developmental and reproductive toxicity

A review of articles on reproductive and developmental toxicity (RT) revealed that the corresponding data sets were modelled thirteen times using machine learning methods. Models were in web services twice (Table 1, Endpoint RT), and one publication [163] had code for model rebuilding in the Supplementary Information. Ten publications presented insufficient information for using their models. It should be noted that the data sets used for modelling were quite large (more than 1000 compounds).

###  Chronic (repeated dose) toxicity

A literature review identified eight publications for chronic toxicity (CT). Most of the found models were tree-based, and although emphasis was put on finding models relevant to human health, half of the articles found described models where aquatic species (e.g., rotifers, algae, water fleas, fish) were used as modelling organisms. The other half used rodents' data. Easy-to-use chronic toxicity regression models for several organisms (algae, water fleas, and fish) were implemented into stand-alone software (Table 1, Endpoint CT). In addition, potentially usable models were found once (Table 2, CT). The remaining six articles did not describe models in a way that they could be usable.

##  Discussion

The reduction of animal testing is an important issue addressed by NAMs. In this context, ML models provide a promising data-driven approach for evaluating chemical hazards in various organisms. Numerous machine learning models have recently been published in scientific literature. This study conducted an exploratory literature analysis of ML models targeting eleven key toxicological endpoints relevant to CRA. Although models were found for each endpoint, the distribution was not uniform. This may be due to certain toxicological endpoints being prioritized in experimental testing, resulting in greater availability of data. Alternatively, it could be because these endpoints are biologically less complex or easier to assess experimentally. Thus, the lack of models for other endpoints was likely due to the lack of available data for model building. It is also possible that some models were not discovered due to search terms that were too general. However, from the user perspective, getting satisfactory results using general terms and filtering out more specific endpoints with additional terms is more helpful.

Other issues were related to the presentation of the models. A more detailed discussion about those shortcomings follows, but in general, these issues relate to the usability and usefulness of models.

###  Usability of models

Creating a machine learning model for a given endpoint to explain processes or make predictions is not a trivial task. Therefore, it is crucial that when published, it is done in a way that models can be easily used and verified. Unfortunately, the results show that the majority of reviewed ML models are published without sufficient details, which makes them unusable on their own. This result is alarming on at least two levels. Firstly, it is impossible to independently verify the results of the research, and secondly, the results of the study (the model) cannot be used by third parties (e.g., risk assessors). Moreover, for risk assessors, as end users, the use and reuse of ML model also requires statistical, programming, and machine learning skills, as well as skills in interpreting and understanding the final prediction results, which may not be the core expertise of many toxicologists. Machine learning-based models have more complex interpretability and often more complex data requirements.

The literature analysis showed that the most straightforward way to make the model usable is to integrate it into a web service or software. While convenient for end users, it may cause problems for model creators. Understandably, many researchers are interested in something other than hosting web services. Web services also hinder the broader integration of models into different systems due to usage policy or data privacy issues. However, publishing models in a standardised model representation file format allows the model to be directly integrated into users’ workflows. Platform-specific formats are helpful if a user is familiar with the platform where the model was created. Cross-platform formats like Predictive Model Markup Language (PMML) or Open Neural Network eXchange (ONNX) were designed to be usable in different ML frameworks and using them can widen the audience that can adapt the model. Additionally, this provides additional assurance that the model can be used even when the web service has stopped working.

PMML is an XML-based markup language for representing and exchanging predictive models across various platforms and applications [164]. It provides a standardised approach for transparently describing model details such as descriptor definitions, data transformations, model representation, and prediction logic, making it valuable for understanding and reproducibility. ONNX [165] is an open format for representing ML models, where the model is described as a computational graph using standardised data types and built-in operators. This approach enables the portability of models across different platforms because the standardised operators are implemented in an external runtime, and each runtime can be optimised for different processor or graphical accelerator architectures.

It is difficult to recommend a single model representation format for all use cases. For many (Q)SAR applications, the PMML format is a good choice because the model representation is text-based and relatively easy to understand, even without any special tools. On the other hand, ONNX is a better option for representing large and complex ML models and Deep Learning models because it uses a binary representation that takes significantly less storage and is quicker to evaluate for predictions. PMML and ONNX formats have an excellent selection of conversion tools for different modelling frameworks and multiple runtime engines for evaluating the models.

From the CRA's point of view, it is essential to publish ML models in a usable and transparent manner. It is a challenging but possible task. However, in some scenarios, it may be sufficient for CRA if it is well described in a scientific publication or has proper documentation in a (Q)SAR Model Reporting Format (QMRF). A model can be represented with cross-platform model formats and made downloadable for added transparency. Making all the scripts and data needed for model creation available is another way to ensure transparency. However, this may sacrifice the usability of the model because the user may need help to recreate the model. Potentially usable models require some effort and skills to be functional. They may also need additional software to standardise and optimise chemical structures and calculate descriptors. This may hinder the usability of those models.

The QsarDB archive format is designed to represent (Q)SAR models [166]. It is a complete solution that can provide a machine-readable representation of the model, together with detailed experimental property data, molecular structure, descriptor data, predictions, and literature references. The QsarDB archive format includes the necessary information for the independent analysis and reproduction of the model. Since it is documented [166] and reuses open data formats, it improves the transparency of models and makes them suitable for long-term data preservation. (Q)SAR model developers can upload their QsarDB archives via the QsarDB repository [167], where their models are made available according to FAIR principles [168] and can be used for data citations via Digital Object Identifier (DOI). The FAIR principles provide essential guidelines for ensuring model sustainability within the scientific community, enabling content discovery, reuse and reproducibility in both research and regulatory assessments. They also encourage machine-readability of models and related (meta)data [169], making them easier to integrate into computational workflows and reducing the risk of obsolescence as time passes and technologies evolve.

Most potentially usable models published code for model recreation, but some were published in the abovementioned formats. Even though code helps reproduce the model, it can lead to a different model due to the stochastic nature of most ML methods. Therefore, storing the final model using the file format designed for it is beneficial. Storing this information in multiple locations is also essential, regardless of the model publishing format.

Searching for models from literature can be tedious, especially when you find promising models, but essential information has gone missing over time. Occasionally, information that should be in Supplementary Information has disappeared or never been published. The same applies to web services. There is no guarantee that they will be present years after publishing. Therefore, the model available in duplicate in different locations, in addition to the web service, is an ideal solution that ensures sustainability.

###  ML model interpretation practices

It is a common belief that the more complex ML models are, the less interpretable they are. That is true, but that does not mean it is impossible. Explainable AI methods [170–172] can help make the results of ML models more transparent and understandable to their users by showing why the particular output was produced, which is important for gaining trust for use in chemical risk assessment [173]. The current analysis also looked at what kind of additional information model developers provided. Most of the effort went into defining the applicability domains of the models, which is an essential aspect of using models. While the models' interpretation of the used descriptors was often left out. However, depending on the model’s complexity, three strategies emerged. Firstly, if the model used a few descriptors, it was possible to connect the information the descriptor gives about the chemical structure to the mechanism of the modelled endpoint. However, modern ML algorithms can handle hundreds of descriptors and connecting them all to the endpoint is not feasible. Some algorithms, like random forest, can determine which descriptors influence the model most. It uses permutation tests to estimate the importance of the descriptors for the model. This is the second approach, where only the most important descriptors are analysed. Lastly, SHapley Additive exPlanations (SHAP) [174] analysis can be performed for any machine-learning model. It can be seen as an extension of the importance analysis. It is a game theoretic approach explaining how each descriptor impacts the prediction. Descriptors with positive SHAP values positively influence the prediction, and negative values have a negative impact. Using this kind of analysis helps to understand whether features of the molecule descriptor illustrate a positive or negative effect on an endpoint.

###  ML models and regulatory compliance gap analysis

Machine learning (ML) models offer promising tools for toxicological assessment, but their integration into regulatory frameworks faces several challenges. Based on the literature reviewed, these obstacles can be broadly summarised as follows:

*Lack of availability and accessibility*: Many models are developed, but their documentation and accessibility are limited.

*Lack of transparency and interpretability*: Many ML models, especially deep-learning models, are considered 'black boxes'. Regulators require clear explanations of how predictions are made and the specific data on which they are based.

*Data quality and standardization*: ML models depend on large, high-quality datasets, but these are often heterogeneous, incomplete, or not standardized, complicating model training and validation as well as clear understanding of the modelled toxicological endpoint.

*Limited validation and reproducibility*: regulatory acceptance requires robust validation across diverse datasets. ML models may perform well on training data but fail to generalise. This aspect should be clearly reported, and the nature of the training dataset used should be explicitly stated.

*Lack of user-friendly interfaces*: Many ML tools are inaccessible to toxicologists or regulators without computational expertise. There is a need for intuitive interfaces and training.

*Binary vs continuous predictions*: Most ML models predict a single binary outcome (e.g., toxic vs. non-toxic), while regulatory decisions often require detailed dose–response relationships for hazard characterization. This is due to the limited availability of experimental dose–response data for the development of the respective QSAR models. Also, for classification models, the cut-off for the classes has to be consistent with what is required for a regulatory decision.

While ML methods that deal with hazard identification are seeing efforts how to document and standardize them [65, 175, 176] regulatory guidelines are not adaptable to AI-based methods (e.g., large language models). Harmonized international standards are needed for validation and reporting. Some important recommendations from the European Food Safety Authority (EFSA) and the European Medicines Agency (EMA) have recently been published. EFSA [177] emphasizes that AI tools can support data extraction and integration from NAMs, but their use must be transparent and scientifically valid. Expert oversight is required at every step, and AI should be seen as a supportive tool rather than a standalone decision-maker. EMA [178] outlines guiding principles for using AI, including governance, accountability, and risk monitoring. These two recommendations, while focused on literature screening and data integration tasks, establish some important principles that are broadly applicable to regulatory contexts of AI, such as transparency, expert oversight, and documented limitations. On the other hand, they do not directly address ML methods or provide technical guidance on ML model validation. However, more specific regulatory guidance documents are needed to meet the ML related requirements.

## Framework for assessing machine learning NAMs

Applying computational NAMs based on modern ML and AI methods is challenging when assessing the properties of chemical substances for hazard identification. Rapid progress in this field is challenging to keep up with, and additional guidance is needed to better exploit these tools in the regulatory context. Guidance is required for model users and developers because the model availability analysis reported above shows that only a small part of published models is usable (17% of analysed publications with models) or potentially usable (28%).

This report describes a framework that guides model users in selecting potentially suitable models for use in a regulatory context. The framework provides a checklist that helps model users quickly evaluate the model and determine whether it includes all the technical information required to use and report results from the model. In addition, the framework could help model developers better understand the expectations of model users in a regulatory context–and potentially also those of model assessors. The model creators can use the framework as a reference when publishing their models, ensuring greater transparency, alignment with regulatory needs, and facilitating future acceptance.

The framework is complementary to the (Q)SAR Assessment Framework (QAF) [179] but has a different scope. The QAF aims to provide a systematic and harmonised framework for the regulatory assessment of (Q)SAR models, predictions, and results based on multiple predictions. The present framework is more general and targets both model users and developers but can also be helpful for risk assessors (e.g., regulatory authorities and their stakeholders). It is designed to be used before QAF and is focused on technical details that are easy and quick to check. The checklist only focuses on critical issues**,** all of which are essential. If some checklist items are not fulfilled, then the model's applicability in the regulatory context is probably impossible. If the model passes the checklist, the assessment with QAF is likely possible because the model is usable, and the necessary information is present for the QAF assessment.

**Fig. 5:**
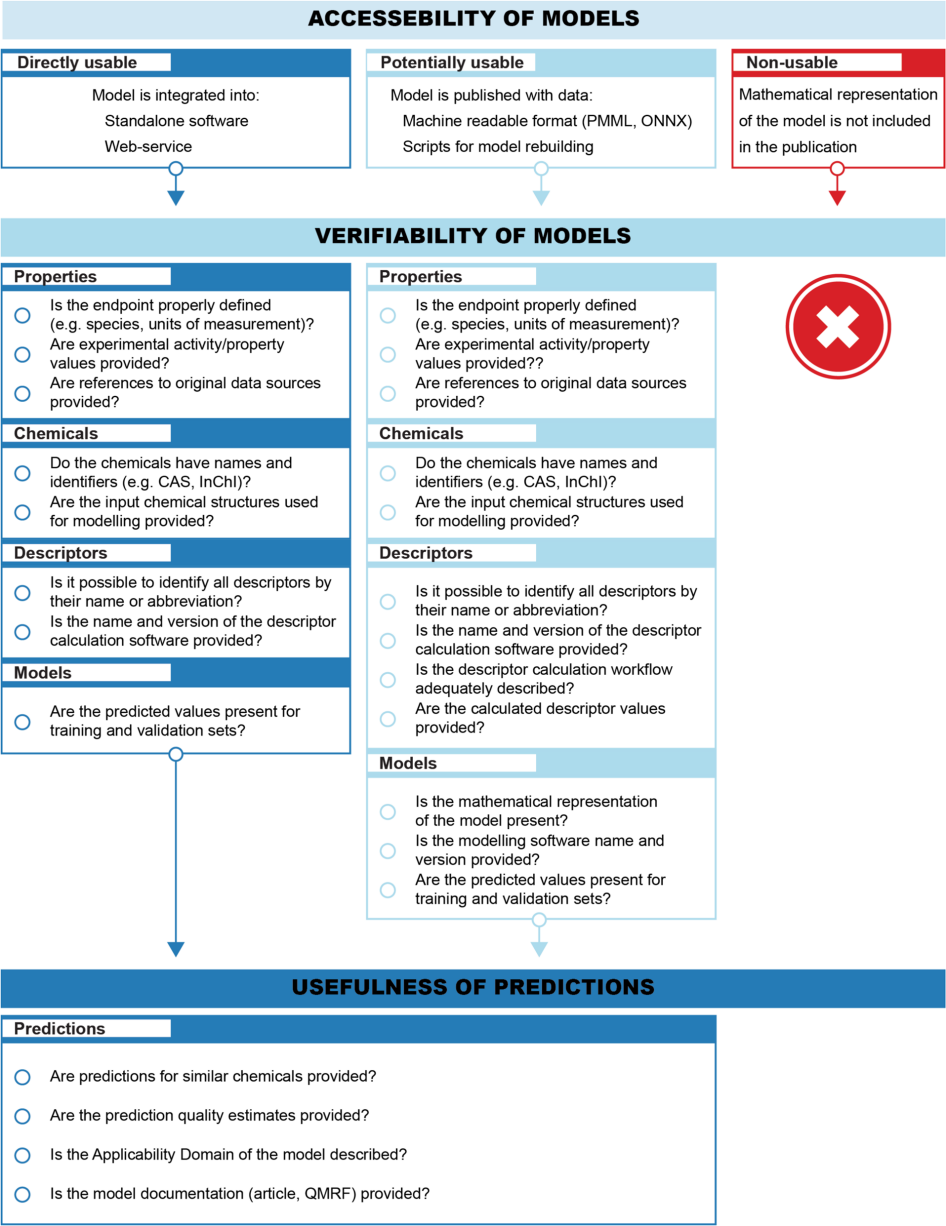
Framework for assessing Machine Learning NAMs

The checklist for the framework (Fig. 5) is presented below, and it captures the minimal requirements needed to reuse the published model. The checklist is organised into three major categories that account for the model accessibility and main subjects in a typical (Q)SAR workflow. All these parts are equally important, and a deficiency in any of them will significantly compromise the usability of models.

### Accessibility of models

The evaluation of the model starts by deciding its accessibility status in terms of the following three questions, and the most appropriate option classifies whether the model is directly usable, potentially usable, or non-usable.

- The Software or Web Service is available (directly usable).
- The publication and data are available, including model representation (e.g., PMML, ONNX) or scripts (potentially usable).
- Mathematical representation of the model is not included in the publication (non-usable).

*Guidance*: This is the most critical part from the point of view of the model users (e.g., regulators, assessors). The published models can be divided into three groups. The first option is ready-to-use software as a standalone software or web service. The second option is a scientific publication with all relevant training and validation data, including the model’s mathematical representation. The second case is potentially usable if the model user has experience and access to the tools the model developers used or can evaluate models represented in machine readable format (e.g., PMML, ONNX). The first case is the most accessible option for the end users, especially for complex ML and AI models. The outcome of the literature review showed that the third option is widespread in the scientific literature, where scientific publications are available without all the necessary data. In this case, such models are unusable and are not worth considering in the framework. These criteria are partly related to the QAF’s model checklist, particularly the assessment element about model accessibility (AE 2.3).

### Verifiability of models

The assessment of the model verifiability is divided into four sections, each focused on a specific part of the model (e.g., property, chemicals, descriptors, and model).

#### Properties

- Is the endpoint properly defined (e.g., species, units of measurements)?
- Are experimental activity/property values provided?
- Are references to original data sources provided?

*Guidance*: This part covers the representation of experimental data. The most important question for the model assessment is whether the experimental data are available. However, the presence of raw data is not sufficient. Defining the measured property (or endpoint) and identifying the original data sources is also necessary. All data manipulations must be described if the data has been processed after being taken from the source. When publishing experimental data, it is advisable to avoid PDF files and prefer machine-readable file formats (e.g., text files such as comma/tab–separated values and spreadsheets). The criteria for evaluating property data are related to the QAF model assessment elements AE 1.1 (clear scientific and regulatory purposes), AE 1.2 (transparency of the underlying experimental data), and AE 1.3 (quality of the underlying experimental data).

#### Chemicals

- Do the chemicals have names and identifiers (e.g., CAS, InChI)?
- Are the input chemical structures used for modelling provided?

*Guidance*: This part of the framework covers the characterization of chemicals and their molecular structures used in model development. It addresses two critical issues related to chemical identification and characterization. First, the correct identification enables the mapping of chemical structures to experimental measurements. It requires the presence of chemical names, Chemical Abstract Service (CAS) registry numbers, International Chemical Identifier (InChI) codes, or other identifiers. The proper characterization of chemicals can be a significant concern for reproducibility because it is not always obvious how to determine the molecular structure of a compound from its chemical identity. Results can vary since different laboratories use different workflows for curating and processing chemical structural data. Discrepancies in the results may arise from different ways of handling salts, mixtures, tautomers, and stereoisomers, or from the generation of 3D coordinates using stochastic algorithms. Therefore, it is strongly recommended that all manipulations conducted during the structure pretreatment are made explicitly available, together with chemical structures in the file format used during modelling. The criteria for chemicals are relevant to the QAF model assessment elements AE 1.2 (transparency of the underlying experimental data) and AE 2.2 (inputs and other options).

#### Descriptors

- Is it possible to identify all descriptors by their name or abbreviation?
- Is the name and version of the descriptor calculation software provided?
- Is the descriptor calculation workflow adequately described?
- Are the calculated descriptor values provided?

*Guidance*: This part covers the descriptors that are used in the model. The availability of descriptor values is critical to verify the descriptor calculation procedure. In addition, the identifiers and names of the descriptors in the article must be consistent with the software used. Identifying the software version(s) used is essential because the descriptor values may change due to bug fixes and improvements to the underlying algorithms. Similarly to the data on experimental properties, it is important to consider the choice of file formats to be used in publishing the descriptor values. These criteria are related to the QAF model checklist (AE 2.1—description of the algorithm and/or software).

#### Models

- Is the mathematical representation of the model present?
- Is the modelling software name and version provided?
- Are the predicted values present for training and validation sets?

*Guidance*: This part covers the representation of the (Q)SAR models and the procedure used for model development. The mathematical representation of the model is essential for performing predictions and understanding how the model works. Complex ML and AI models must be represented in a machine-readable format (e.g., PMML, ONNX). Alternatively, another option is providing scripts for reproducing models from the training data. The description of the model development process must contain the name and version of the modelling software, together with the parameter values specific to the modelling technique used to build the model. In addition, the availability of the predicted property values is important because they enable the verification of each prediction when model reproduction is attempted. Also, the predicted values are important for validating the model in global terms by allowing an independent verification of the published model statistics or the calculation of additional statistical metrics. When the model is available as a ready-to-use software or web service, the first question about the model representation is not important for performing predictions; however, the last question is critical for independent verification that the model is working correctly. These criteria are related to the QAF model checklist via AE 2.1 (description of the algorithm and/or software) and AE4.1 (goodness-of-fit, robustness).

### Usefulness of predictions

The evaluation of prediction usefulness assumes that the checklist above has been passed, and this part is only focused on supplementary information that is provided with the model predictions.

- Are predictions for similar chemicals provided?
- Are the prediction quality estimates provided?
- Is the Applicability Domain of the model described?
- Is the model documentation (article, QMRF) provided?

*Guidance*: This part focuses on the requirements for the prediction results from the model that make it useful for decision-making. The list of questions in this category is not exhaustive and only includes the most valuable options for consideration. The first two questions address the reliability of the prediction. For example, if the prediction includes predictions for similar chemicals in the data set, then it helps assess the local predictivity of the model. Similarly, providing prediction quality estimates (e.g., confidence intervals, probability) helps to determine the uncertainty associated with the prediction. The remaining criteria (i.e., applicability domain description and model documentation) are important requirements for the regulatory assessment of the model and its predictions. Although ideally, it should be provided by the model developers, it may be possible to make an independent assessment if the model passes the accessibility and verifiability parts of the checklist above. These criteria are related to the QAF model checklist via AE 2.2 (inputs and other options) and AE 3.1 (a defined domain of applicability).

##  Conclusions

Data-driven machine learning has become an important part of computational NAMs, and machine learning computational models are emerging as an important tool for identifying chemical hazards. This study reviewed the scientific literature to determine the availability and applicability of machine learning models for hazard identification in the CRA framework. The study included recently published computational NAM models targeting various human health endpoints, such as specific target organ toxicity (STOT), genotoxicity and carcinogenicity, endocrine disruption, skin sensitization, developmental and reproductive toxicity (DART), and repeated/chronic toxicity.

As a result of the study, nearly 2300 scientific articles were reviewed, and 274 publications with published ML-QSAR models were identified, i.e., where the development of such models has been most intensive. These models were classified as directly usable, potentially usable, or non-usable based on published information. The study unfortunately revealed that 60.9% of the models described in the scientific literature turned out to be non-usable, 21.9% were potentially usable, and 17.2% were usable, i.e., had available software solutions. By endpoint, the skin sensitization is best covered with the ML-QSAR models, followed by models for endocrine disruption, genotoxicity, and carcinogenicity. The least covered are specific target organ toxicity endpoints. The most derived ML-QSAR models are tree-based models, such as random forests, followed by artificial neural networks and support vector machine models, with others being used to a lesser extent.

The practical applications of computational NAMs based on modern machine learning and artificial intelligence methods are a challenging task when assessing the properties of chemical substances for hazard identification. With the rapid progress in this field, it is difficult for both model developers and, even more so, for model users to keep up. Therefore, more guidance is needed to help better development and application of these methods in a regulatory context. To respond to these challenges, the OECD has recently published the QAF for regulators and their stakeholders, which provides a systematic and harmonized framework for evaluating the validity and reliability of predictive models and predictions. While this framework is general and flexible across different modelling approaches, there is a lack of knowledge and experience on how to use it with modern ML or AI methods. Considering all the major groups of participants, academia, industry, regulators, the obvious challenge is to overcome the internal "contradictions" or "barriers" between them, which on the one hand would help to increase confidence in machine learning approaches, demystify them, and on the other hand would show the need to understand the technical content, through training, which is the key to a better understanding. Thus, frameworks and collaboration platforms that bring together different knowledge and usage are of great help here.

This literature review and the associated work have led to the creation of a relatively simple and transparent framework. It aims to help model developers and users assess whether a model meets the minimum essential criteria for consideration in regulatory applications. The framework provides a checklist that enables model users to quickly evaluate a model and determine whether it contains all the technical information necessary to use it for prediction and reporting. It also supports model developers by clarifying the essential criteria their models should meet when published, promoting consistency, transparency, and regulatory readiness.

## Supplementary Information

Supplementary Material 1.

## Data Availability

No datasets were generated or analysed during the current study.

## Abbreviations

AI: Artificial intelligence
AOP: Adverse outcome pathway
ANN: Artificial neural networks
CRA: Chemicals risk assessment
DA: Defined approach
DART: Developmental and reproductive toxicity
DL: Deep learning
DNT: Developmental neurotoxicity
DT: Decision tree
ECHA: European chemicals agency
EFSA: European food safety authority
EMA: European medicines agency
EU: European union
FAIR: Findable, accessible, interoperable, reusable
IATA: Integrated approaches to testing and assessment
ICCVAM: Interagency coordinating committee on the validation of alternative methods
ML: Machine learning
NAM(s): New approach methodologies
OECD: Organisation for economic co-operation and development
ONNX: Open neural network exchange
PARC: Partnership for the assessment of risks from chemicals
PBK: Physiologically based kinetic
PMML: Predictive model markup language
QAF: (Q)SAR assessment framework
QMRF: (Q)SAR model reporting format
QSAR: Quantitative structure–activity relationship
QsarDB: QSAR database
qAOP: Quantitative adverse outcome pathway
RF: Random forest
SAR: Structure–activity relationship
SHAP: SHapley additive explanations
STOT: Specific target organ toxicity
SVM: Support vector machine
UK CoT and FSA: United Kingdom, Committee On Toxicity and Food Standards Agency
US EPA: United States Environmental Protection Agency
XML: EXtensible markup language
